# Epigenetic Modifications in Osteosarcoma: Mechanisms and Therapeutic Strategies

**DOI:** 10.3390/life15081202

**Published:** 2025-07-28

**Authors:** Maria A. Katsianou, Dimitrios Andreou, Penelope Korkolopoulou, Eleni-Kyriaki Vetsika, Christina Piperi

**Affiliations:** 1 Department of Biological Chemistry, Medical School, National and Kapodistrian University of Athens, 11527 Athens, Greece; mairakatsianou@gmail.com (M.A.K.); andreou_dimitris@hotmail.com (D.A.); 2 First Department of Pathology, Medical School, National and Kapodistrian University of Athens, 11527 Athens, Greece; pkorkol@med.uoa.gr; 3 Centre of New Biotechnologies and Precision Medicine (CNBPM), School of Medicine, National and Kapodistrian University of Athens, 11527 Athens, Greece; ekvetsika@med.uoa.gr

**Keywords:** osteosarcoma, epigenetic mechanisms, biomarker, DNA methylation, non-coding RNAs, therapeutic targets, AI-based therapies

## Abstract

Osteosarcoma (OS), the most common primary bone cancer of mesenchymal origin in children and young adolescents, remains a challenge due to metastasis and resistance to chemotherapy. It displays severe aneuploidy and a high mutation frequency which drive tumor initiation and progression; however, recent studies have highlighted the role of epigenetic modifications as a key driver of OS pathogenesis, independent of genetic mutations. DNA and RNA methylation, histone modifications and non-coding RNAs are among the major epigenetic modifications which can modulate the expression of oncogenes. Abnormal activity of these mechanisms contributes to gene dysregulation, metastasis and immune evasion. Therapeutic targeting against these epigenetic mechanisms, including inhibitors of DNA and RNA methylation as well as regulators of RNA modifications, can enhance tumor suppressor gene activity. In this review, we examine recent studies elucidating the role of epigenetic regulation in OS pathogenesis and discuss emerging drugs or interventions with potential clinical utility. Understanding of tumor- specific epigenetic alterations, coupled with innovative therapeutic strategies and AI-driven biomarker discovery, could pave the way for personalized therapies based on the molecular profile of each tumor and improve the management of patients with OS.

## 1. Introduction

Osteosarcoma (OS) is a rare mesenchymal malignant tumor and represents one of the most common cases of pediatric and adult tumors. It occurs during the growth stages of long bones due to failure in osteogenesis surveillance, allowing genetically altered cells to survive and produce malignant osteoid [1]. It is characterized by widespread and heterogenous abnormalities in chromosomal number and structure, unlike other sarcomas [2]. The frequency of this neoplasm in the general population is estimated to be 2–3 cases/million in the first year in children and rising up to 8–11/million in the age groups of 10–19 years old, along with poor prognosis in adolescents [3]. Among patients diagnosed with primary OS, 65% achieve a 5-year disease-free survival (DFS), whereas the survival rate drops below 30% in those with metastatic disease [4].

OS is characterized by osteoid production and a woven bone matrix, which varies from dense, well-formed trabeculae to inconspicuous amounts. Histological evaluation has revealed several subtypes in OS classification, including osteoblastic, fibroblastic, chondroblastic and telangiectatic, with the first three referred to as osteogenic sarcomas, and telangiectatic sarcoma, which is considered an osteogenic sarcoma, but a more aggressive, high-grade subtype with worse prognosis [5,6]. Despite their histological differences, OS subtypes are biologically similar, marked by genome disorganization, aneuploidy, absence of DNA repair mechanisms and chromosomal alterations [5]. Thus, the molecular and epigenetic profiles remain key determinants of OS pathogenesis across subtypes.

The most known genetic alterations underlying OS pathology involve mutations in genes regulating cell cycle and apoptosis, with *p53* [7], tumor suppressor *Rb* (retinoblastoma) [8], *Runx2* [9] and *c-Myc* [10] being the main genes that contribute to disease progression in OS [2,11]. Many syndromes are also associated with OS, like Bloom syndrome (with a germline mutation of the *RECQL2* gene), Werner syndrome (*RECQL3* gene mutation) and Rothmund–Thomson syndrome (*RECQL4* gene mutation). Somatic mutations further modify these genes and the respective signal transduction pathways in OS; however, the mechanisms inducing these mutations are distinct from these germline mutations [12].

**Figure 1:**
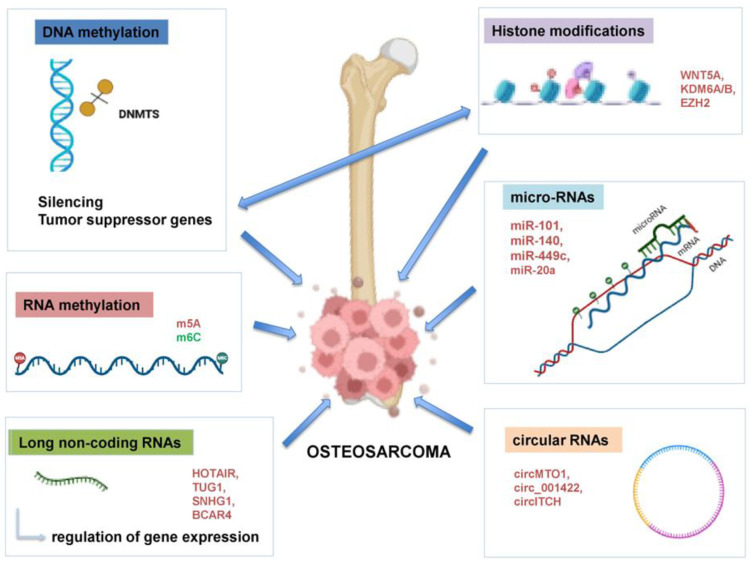
Major epigenetic mechanisms implicated in OS pathogenesis. DNA methylation, histone modifications, non-coding RNA regulation, RNA methylation and chromatin remodeling interconnected processes alter gene expression and chromatin, driving tumor initiation, progression, metastasis and therapy resistance. DNMT: DNA methyltransferase; HIF-1α: Hypoxia-Inducible Factor 1-Alpha; GADD45: The Growth Arrest and DNA Damage-inducible 45; HIC-1: Hypoxia-inducible factor-1; IRX1: Iroquois-class homeodomain protein; HOTAIR: HOX Transcript Antisense RNA gene; TUG1: taurine upregulated gene 1; SNHG1: Small Nucleolar RNA Host Gene 1; BCAR4: Breast Cancer Anti-Estrogen Resistance 4; WNT5a: Protein Wnt-5a, KDM6A/6B: Lysine Demethylase 6A/6B; EZH2: Enhancer of zeste homolog 2; miR: microRNA; circ: circular RNA. Created in https://BioRender.com (accessed on 19 July 2025).

DNA methylation, histone modifications and nucleosome remodeling of ncRNAs in OS development are epigenetic modifications and possible risk factors for OS progression, serving as potential biomarkers for prognosis and therapeutic targets (Figure 1) [13]. Experimental studies indicate that the activity of tumor suppressor genes or p53 protein-targeted genes is often suppressed due to promoter hypermethylation in OS-derived cell lines, with osteoblasts being able to differentiate into multiple cell types in osteogenesis through complicated signaling pathways. Thus, while OS disease progression is attributed to mutations in genes regulating the cell cycle and apoptosis, epigenetic changes often co-exist, serving either complementary or independent roles. Promoter hypermethylation and histone modifications may act as secondary mechanisms that silence tumor suppressor pathways when genetic mutations impair canonical ones. Conversely, epigenetic dysregulation may arise independently, initiating oncogenesis by disrupting gene expression in the absence of known mutations. Therefore, the origin of OS is considered as a complex multifactorial event that involves the interplay of oncogenes, tumor suppressor genes, cytokine dysregulation and epigenetic mechanisms to mediate the malignant phenotype of tumors [14].

Recent advances in OS research have stimulated numerous studies to identify prognostic biomarkers and evaluate corresponding risk models by delving deeper into gene sequencing and tumor-specific epigenetic modifications [13]. However, clinical trials are currently deemed challenging due to the enforced epigenetic DNA alterations and the unpredictable expression of oncogenes that may lead to tumor development. Studies have revealed that epigenetic changes in OS occur in a three-dimensional environment, with various factors influencing tumor cell proliferation and cancer progression [14], which is not fully reflected in in vitro models. Lin et al. developed a three-dimensional (3-D) bioprinted model comprised of OS cells and an analogue of the surrounding extracellular matrix. DNA methylomics and KEGG pathway (Kyoto Encyclopedia of Genes and Genomes) analysis in this model revealed different methylation states in the 3-D model cells versus actual OS cells [15]. Therefore, these findings suggest that 3-D models can simulate the tumor microenvironment and its epigenetic landscape, providing a physiologically relevant basis for investigating OS biology. Such models can be more efficient compared to oversimplified 2-D systems for drug screening and for assessing epigenetic therapies. Specific amino acids, including valine, isoleucine and leucine degradation, along with the analysis of the most enriched pathways, demonstrated a significantly altered methylation state. The multi-omic analysis identified 276 genes that distinguished two categories of differentially expressed genes (DEGs) and differentially methylated positions (DMPs) between the 2-D plates and the 3-D model [15]. Additional studies on histone modifications revealed that histone demethylation in OS, along with reduced levels of OS histone methylation, is attributed to diminished levels of histone methyltransferases [13]. A significant loss of histone 4 lysine 20 trimethylation (H4K20me3) has been associated with poor prognosis in various human cancers, including OS. Studies in non-human OS cases have further detected histone methylation to be a contributing factor, along with several mutations present in canine OS across all breeds, using whole-exome sequencing [16]. Recently, non-coding RNA (ncRNA) has garnered attention in cancer research due to its substantial epigenetic role in altering gene expression in tumor cells [17]. Furthermore, the identification of specific epigenetic modifications linked to OS not only enhances our understanding of its pathophysiology but also contributes to the possibility of developing epigenetic therapies that could reverse or inhibit these modifications. 

In this review, we provide an update on the epigenetic mechanisms underlying OS pathology, focusing on their implications in disease progression and potential use as targets for novel therapeutic approaches. We also explore recent advances in epigenetic-based therapies, highlighting the challenges and future directions for enhancing treatment efficacy and clinical outcomes in OS.

## 2. Epigenetic Mechanisms in Cancer

Epigenetics refers to chemical modifications in DNA and chromatin which do not cause alterations to the underlying nucleotide sequence but regulate gene expression and function [18], with certain epigenetic modifications being related to heredity factors [19]. The epigenome—the collection of all epigenetic changes in the genome—is formed by genetic and environmental factors, mostly three epigenetic mechanisms: DNA methylation, histone modification and nucleosome remodeling (Figure 1) [20].

DNA methylation refers to the addition of a methyl group to the C5 position of cytosine, within CpG islands in gene promoters, generating a 5-methylcytosine. It is a crucial event implicated in the processes of genomic imprinting, X-chromosome inactivation and suppression of transposable elements [21]. CpG islands, present in 70% of gene promoters, can regulate gene silencing through hypermethylation, one of the most renowned mechanisms of dysregulation in cancer [19,22]. The CpG methylation patterns change dynamically during development [23]. DNMT1 maintains the methylation levels of DNA, while DNMT3a and DNMT3b mediate de novo methylation when the organism is in the state of embryogenesis, establishing genomic imprints. Aberrant hyper- or hypomethylation contributes to genome instability and tumor development [21]. In OS studies, aberrant methylation of promoter regions leads to silencing of tumor suppressor genes such as *RASSF1A* and *p16*, contributing to uncontrolled growth, tumor progression and unfavorable prognosis [24,25].

Histone modifications such as phosphorylation, methylation, acetylation and ubiquitination are post-translational modifications on the N-terminal of histones in nucleosomes affecting chromatin’s structure and regulating gene transcription [26,27]. These histone modification patterns have been assigned as the “histone code” that is believed to co-ordinate chromatin accessibility, gene expression and cell growth [28]. Irregular acetylation with histone modifications promotes aberrant oncogene expression and silencing of tumor suppressor genes, which subsequently leads to tumorigenesis [29]. Dysregulated histone demethylase activity, particularly of KDM5B, as well as diminished H4K20me3 levels have been observed in OS and are associated with increased tumor aggressiveness and resistance to chemotherapy, suggesting their involvement in tumor progression and drug resistance [30].

Nucleosome remodeling modulates and changes the positions of the nucleosomes, altering the interaction of DNA with histones and disrupting potential binding of transcription factors to DNA, by ATP-dependent chromatin-remodeling complexes (CRCs) [31]. These complexes are involved in transcriptional regulation during crucial cellular processes such as DNA replication, DNA damage and specification of cellular identity. Dysregulation of these processes can lead to tumorigenesis or cellular transformation [32]. Alterations in the expression of SWI/SNF components in OS disrupt chromatin remodeling that epigenetically silences tumor suppressor genes, highlighting their role in disease progression and potential targeting in epigenetic therapy [33,34].

In addition, ncRNAs, although they cannot translate into proteins, perform crucial regulatory functions within the cell, enabling gene expression or suppression, and play an essential role in epigenetic regulations in cancer [35]. These include long non-coding RNAs (lncRNAs), micro-RNAs (miRNAs) and circular RNAs (circRNAs). lncRNAs regulate imprinting and X-chromosome inactivation, and they can also affect transcription by other mechanisms, such as the use of RNA-binding proteins or prohibiting association with *TFIID* (Transcription Factor II D) promoter [36]. They also regulate signaling pathways or interact with miRNAs to regulate signaling and gene expression. Studies have shown that lncRNAs can promote oncogenesis, acting either as suppressors or as oncogenes, initiating tumor development [37,38]. circRNAs present a distinct class of endogenous ncRNAs formed by back-splicing, act as miRNA sponges and control crucial biological processes such as transcriptional or post-transcriptional expression [39]. Studies in OS demonstrate that specific ncRNAs, such as lncRNA MALAT1 and circRNA hsa_circ_0001658, contribute to epigenetic regulation of tumor growth and metastasis through modulation of key oncogenic pathways such as Wnt/β-catenin and PI3K/AKT [40].

Accumulating scientific evidence highlights the significant contribution of epigenetic mechanisms in the transcriptional gene dysregulation observed in human OS, and are discussed in more detail in the following sections.

## 3. Implication of Epigenetic Mechanisms in OS

A deeper understanding of the epigenetic changes underlying OS pathogenesis is crucial to advance our knowledge of OS biology, cancer onset and future targeting. In this section, we review the major epigenetic modifications implicated in OS, with emphasis on recent advances in the field and future perspectives (Figure 1).

### 3.1. Tumor Suppressor Pathways Affected by Methylation

DNA methylation is a pivotal epigenetic alteration in OS, affecting key tumor suppressor pathways, oncogenes and the broader regulatory landscape of gene expression. The main tumor suppressor pathways affected by methylation include p53 (tumor protein 53) and Rb (retinoblastoma protein). P53, a central regulator of gene transcription and cell fate decisions, mediates processes such as DNA repair, apoptosis, cell cycle arrest and autophagy triggered by hypoxia, toxins and DNA damage [41]. While TP53 mutations are common in OS, components of its regulatory network are also subjected to epigenetic silencing. For instance, *CDKN2A* (encoding p14ARF) is hypermethylated in up to 47% of OS tumors and cell lines, leading to impaired p53 stability and function [42,43]. Similarly, the *p21* gene, which is regulated by *p53* to block G1-to-S cell cycle transition, was shown to be restored by decitabine, indicating that *p21*, like *p14ARF*, can be suppressed by DNA hypermethylation [44]. The loss of *p53* function is considered as a primary genetic defect in osteosarcoma; however, many factors within the p53 pathway, including p14ARF, are silenced by promoter hypermethylation in both OS cell lines and 47% of tumor samples [42,43]. HIF-1 (Hypoxia-inducible factor-1) is overexpressed in OS tissues and cell lines, promoting cell proliferation, migration and invasion and suppressing apoptosis. HIF-1 can also interact with the AKT pathway, indicating a positive feedback loop that may further disrupt p53 activity [45].

The retinoblastoma (Rb) pathway is another critical cell cycle control regulator at the G1 phase and cell differentiation. While *Rb* itself is rarely inactivated by promoter methylation in OS, epigenetic silencing of key pathway members has been documented [46]. For instance, cell cycle-regulating proteins such as p16INK4a (encoded by the *CDKN2a* gene) and CREG1 (Cellular Repressor of E1A-Stimulated Genes 1), that regulates cell growth and differentiation, have been shown to undergo promoter hypermethylation, leading to reduced gene expression.

### 3.2. DNA Methyltransferases (DNMTs)

Aberrant activity of DNA methyltransferases (DNMTs) has been associated with OS pathogenesis, altering gene expression through DNA methylation as previously described. Among them, DNMT1 is frequently overexpressed in OS tissues in comparison to normal cells [4] and promoter methylation of miR-34a by DNMT1 promotes stemness in OS stem-like cells. Similarly, miR-139-5p can target DNMT1 and inhibit osteosarcoma growth, with its overexpression reducing tumor growth in vivo [47]. DNMT3B also contributes to OS development since its copy number variants and overexpression are related to CpG island hypermethylation [48]. A pediatric cancer study revealed 139 genes with variable methylation. Among them, *Spry2* showed the greatest change and exhibited a 2.9-fold increase in methylation in OS compared to clear cell sarcoma of the kidney [49]. Such findings highlight the need for further research in establishing the DNMTs’ biomarker potential for diagnosis and therapy in OS.

### 3.3. Additional Methylation Targets

*GADD45* (The Growth Arrest and DNA Damage-inducible 45) regulates critical processes such as cell cycle control, DNA repair, senescence and apoptosis in response to genotoxic stress. In osteosarcoma, hypermethylation of the *GADD45* promoter has been detected in cell lines, leading to its epigenetic silencing [50]. This loss of function impairs DNA damage responses and promotes tumorigenesis.

Additionally, studies have reported that 17–77% of OS cases exhibit hypermethylation of the *HIC1* (Hypermethylated in Cancer 1) gene promoter. HIC1 is a critical regulator of p53-dependent responses to DNA damage and an important contributor to induced regulatory T cell development and function. Silencing of *HIC1* results in the activation of SIRT1 (Sirtuin 1), a key deacetylase that acts on transcription factors involved in cellular regulation, leading to the suppression of *p53* [44].

**Figure 2:**
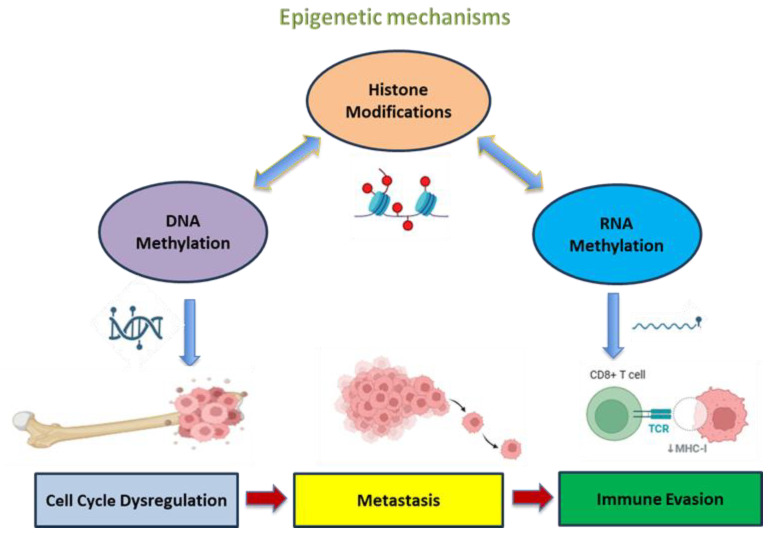
Epigenetic crosstalk in OS progression. The crosstalk between DNA, RNA methylation and histone modifications induces epigenetic changes that activate oncogenic pathways, leading to cell cycle dysregulation, metastasis and immune evasion, enhancing tumor progression. Created in https://BioRender.com (accessed on 19 July 2025).

A study on metastatic OS identified the *IRX1* gene (Iroquois homeobox protein 1) as a potential pro-metastatic factor upon methylation. *IRX1* encodes a member of the iroquois homeobox protein 1 family, which plays a role in pattern formation in both vertebrate embryos and invertebrate species and has been implicated in suppression of gastric cancer [51]. Hypomethylation of the *IRX1* promoter leads to its overexpression in OS and is further associated with increased pulmonary metastasis. The pro-metastatic effects of IRX1 are possibly attributed to the positive modulation of the CXCL14/NF-κB signaling pathway, promoting migration and invasion in vitro and in vivo, respectively (Figure 2) [52]. Additionally, *HOTAIR* (HOX Transcript Antisense RNA), a critical factor in the epigenetic regulation of skin differentiation, is highly expressed in OS cells. Its repression leads to downregulation of DMNT1, leading to an overall reduction of DNA methylation levels. Silencing of HOTAIR causes increased expression of DNMT1, leading to hypermethylation of the *CDKN2A* promoter and subsequent gene silencing, which contributes to tumorigenesis. Other tumor suppressor genes, such as *RASSF1A, TIMP3, MGMT, DAPK1* and *WIF-1*, have been revealed to be affected by hypermethylation in OS studies [53].

A detailed analysis of 1.1 million methylation sites in a study of OS biopsy samples showed that 6.6% of patients with relapsed OS exhibited promoter hypermethylation, and 2% showed promoter hypomethylation, while less than 1% of patients without relapse exhibited high methylation [54]. This study indicates the potential predictive value of DNA methylation information in pediatric osteosarcoma outcomes.

### 3.4. RNA Methylation

RNA methylation activates downstream protein “readers” that recognize the methyl group, contributing to the stability, splicing and translation of the methylated mRNA or nuclear export depending on YTHDF1/2/3 and YTHDC1/2 reader proteins. Specifically, N6-methyladenosine (m6a) is the most common mRNA modification that leads to altered gene expression, mediated by methyltransferase-like proteins (METTLs) [55]. Abnormal m6A methylation patterns found in various cancers affect tumor progression and the tumor microenvironment (Figure 1), being often linked to methyltransferase-like 3 (METTL3) activity. METTL3 is upregulated in OS and, by increasing the levels of m6A on certain oncogenes’ mRNA, it stabilizes their transcripts and increases the efficiency of their translation [56]. More specifically, m6A methylation of *MYC* and *ATAD2* mRNAs was shown to stabilize their transcripts through YTHDF1/2 binding, elevating protein expression and enhancing proliferation [57]. MTTL3 has been associated with the overexpression of the *DRG1* (developmentally regulated GTP binding protein 1) gene, which promotes tumor growth and metastasis in OS. m6A levels are critical in embryonic development but they are downregulated after birth and can be affected by signals such as DNA damage, hypoxia and homocysteine levels [58]. Loss of METTL3 impairs translation of tumor-suppressive genes, enhancing NF-κB and STAT3 [58,59].

5-methylcytosine (m5C) modifications that also occur in RNA molecules may influence osteosarcoma progression, enhancing mRNA stability and increasing translation through interaction with ALYREF or YBX1 reader proteins. The m5C methyltransferase NSUN2 is often elevated in osteosarcoma [60]. It can mediate m5C on hepatoma-derived growth factor (HDGF) or VEGF mRNAs, resulting in increased stability and inducing angiogenesis and metastasis. In accordance with this, the reader YBX1 binds and stabilizes oncogenic transcripts, promoting efficient translation in OS [60,61]. A recent study has revealed that an m5C-based risk score (including *C1orf64, AKT1S1* and *XRCC6*) has higher prognostic efficacy than previous models and is linked to methylation regulators (MBD2, UHRF2 and TET3), focal adhesion pathways and immune cell infiltration [62].

### 3.5. Histone Modification in OS

Tumor cells generally exhibit hypoacetylation compared to healthy cells. Rb regulates acetylation and histone methylation through interactions with histone-deacetylating and -methylating complexes, modulating chromatin structure. Recent studies have shown that osteosarcoma cells deficient in both *Rb* and *p53* can differentiate into osteoblastic and adipogenic lineages, whereas *p53*-deficient cells can differentiate into bone or fat cells [63]. This evidence indicates that Rb-mediated modifications to chromatin are critical in determining the differentiation potential of osteosarcoma cells. Moreover, the lysine-specific demethylase 1 (LSD1) was detected at high levels in OS, and treatment with the inhibitor tranylcypromine was shown to result in decreased cell growth.

Histone modifications, along with DNA methylation, regulate gene expression or induce gene silencing (Figure 2), depending on the specific amino acid residue modified. For example, H3K4, H3K36 and H3K79 methylations are considered as activation marks, enabling gene transcription, while H3K27 and H4K20 methylations are repressive marks, inducing gene silencing [64].

The *WTN5A* (Wnt Family Member 5A) gene, which encodes a signaling glycoprotein and its mutation, has been associated with various tumor types [64]. *WNT5A* promoters A and B were investigated in the U2OS and SaOS-2 OS cell lines, as well as in tumor tissues and normal osteoblasts. A and B promoters in normal osteoblasts are active, with promoter B being 10 times more active than promoter A. On the other hand, exon 1β of promoter B, which contains three enriched CpG islands, is hypermethylated in both U2OS and SaOS-2 cells. The regulation of promoters A and B has been proposed to be regulated by histone modifications in OS pathogenesis. Specifically, the histone activation mark H3K4me3 was overexpressed in promoter A and repressed in promoter B, while the repressive mark H3K27me3 was enriched in promoter B of SaOS-2 cells. Thus, repressed WNT5A expression in osteosarcoma cells may result from epigenetic mechanisms, both DNA methylation and histone post-translational modifications. Moreover, *KDM6A* and *KDM6B* genes (lysine demethylases) after cisplatin treatment are involved in H3 lysine demethylation (H3K27me3) in OS cells. EZH2 methyltransferase was further shown to decrease the levels of H3K27me3 in OS cells, while inhibition of KDM6A or KDM6B demethylases enhanced H3K27me3 levels, thus regulating cisplatin resistance and influencing the effectiveness of cisplatin in OS therapy [65].

The deacetylases HDAC1,2 are also overexpressed in OS cell lines and tissues, suppressing *p21* and *RUNX3* levels via H3K27 and H3K9 deacetylation, leading to increased proliferation and apoptosis resistance as well as chemoresistance [66,67]. Additionally, the histone acetyltransferase p300 has been shown to mediate *MYC* acetylation, promoting its transcription. The methyltransferase SETD2 that establishes the active mark H3K36me3 is frequently mutated in OS, leading to tumor suppressor gene silencing [68]. Accordingly, DOT1L methyltransferase has been shown to promote transcription of *c-MYC* and *HOXA9* genes, associated with cell proliferation and stemness [69]. Another histone lysine methyltransferase, SUV39H1, is upregulated in OS, establishing repressive H3K9me3 in *CDKN2A* and *TP53* genes, inducing their silencing [70]. Finally, the reader of acetylated histones, BRD4, is often overexpressed in OS, upregulating *MYC* and *BCL2* gene expression and promoting cell survival.

### 3.6. MicroRNAs (miRNAs)

MicroRNAs, a class of small noncoding RNAs comprising 20–30 nucleotides, play a fundamental role in the cell cycle, embryonic development, immunity and apoptosis. They are also important in targeting and silencing numerous genes involved in OS signaling pathways such as Ras, Wnt, Notch and mitogen-activated kinase (MAPK), as well as DNMTs [26,71].

Dysregulation of miRNA expression results in the dysregulation of gene expression in OS, leading to uncontrolled tumorigenesis [72]. Various miRNAs have been reported as dysregulated due to epigenetic alterations in OS, including miR-16, miR-113a, miR-143, miR-34, miR-199a-3p, miR-335 and miR-340 (Table 1). In comparison, some miRNAs are overexpressed in OS, such as miR-9, miR-21, miR-27a, miR-21 and miR-221. Notably, miR-9 appears to promote osteosarcoma cell proliferation and its inhibition reduces migration and invasion in OS cell lines. Similarly, miR-27a upregulation in OS has been associated with increased tumor aggressiveness and poor prognosis [73]. miR-21 is also considered as an oncogene, inhibiting apoptosis, presenting biomarker potential for osteosarcoma diagnosis and prognosis, as well as a promising therapeutic target [74]. Overexpression of miR-221 and miR-222 has been associated with OS development and progression, since they can promote proliferation and contribute to cisplatin resistance [75]. Furthermore, miR-20a has been implicated in the epigenetic downregulation of Fas, while miR-140 targets histone deacetylase 4 (HDAC4) [76,77]. In addition, a clinical study consisting of 85 patients with resectable tumors and 56 with unresectable OS revealed that lower miR-125b expression was associated with a shorter disease-free survival period and poorer prognosis, especially in unresectable cases. Bioinformatics analyses revealed that miR-140 targets HDAC4, acting as a tumor suppressor. Restoration of miR-140 expression reduced metastasis and enhanced cell death [78]. However, there is limited knowledge on epigenetic mechanisms causing this miRNA overexpression.

In MG-63 OS cells, overexpression of miR-101 suppresses *ROCK1*, a gene that promotes cellular invasion and migration, promoting apoptosis. miR-101 blocks the JAK/STAT and PI3K/AKT signaling pathways, further reducing OS motility [79]. Similarly, miR-3928 inhibits tumor growth and induces apoptosis in OS cells by targeting *CDK6*, *IL-6R* and *ERBB3* genes, which encode the cyclin-dependent kinase 6, IL-6 receptor and tyrosine protein kinase receptor [52].

On the other hand, several miRs downregulated in OS affect metastasis (e.g., miR-143, miR-34a, miR-192 and miR-200 family) and therapy resistance (e.g., miR-34a, miR-133b, miR-140 and miR-29 family) (Table 1). In the metastatic cell line LM7, the microRNAs miR-20a and miR-19a were linked to the reduced expression of Fas protein, a critical member of the TNF receptor family, involved in immune regulation and apoptosis. Inhibition of these miRNAs restored Fas functionality and significantly reduced the levels of metastasis in both cell models and mouse experiments [80]. Finally, the hypermethylation of two neighboring CpG islands led to the epigenetic silencing of miR-449c. Expression of miR-449c further suppressed the oncogene *c-Myc* activity and inhibited colony formation in OS cells [81].

**Table 1:** miRNAs implicated in OS metastasis and therapy resistance.

miRNA	Expression	Target	Effect in OS	Mechanism	Reference
miR-9	Elevated	E-cadherin	Loss of epithelial characteristics	Induces EMT and lung metastasis	[82]
miR-21	Elevated	PTEN, PDCD4	Activates Akt pathway	Apoptosis resistance and proliferation	[74,83]
miR-27a	Elevated	FOXO1	Promotes cell migration	Induces invasion	[73]
miR-221/222	Elevated	p27, TIMP3	Promotes survival and cell cycle progression	Resistance to cisplatin and methotrexate	[75,84]
miR-29 family	Reduced	MCL-1, COL1A1/2	Enhances ECM and anti-apoptotic proteins	Broad resistance to chemotherapy	[85]
miR-34a	Reduced	c-MET, CDK6, BCL2	c-MET promotes motility and EMT, inhibits apoptosis when downregulated	Induces migration and lung metastasis. Resistance to doxorubicin and cisplatin	[86]
miR-133b	Reduced	MDR1/P-gp	Increases efflux pump expression	Multidrug resistance	[87]
miR-140	Reduced	HDAC4	HDAC4 promotes survival signaling	Cisplatin resistance	[77,78]
miR-143	Reduced	MMP-13	Promotes ECM degradation	Induces invasion and metastasis	[77]
miR-192	Reduced	TGFβ1	TGFβ pathway upregulated	Promotes EMT and metastasis	[88]
miR-200 family	Reduced	ZEB1/ZEB2	Promotes EMT via E-cadherin suppression	Induces metastasis	[89]

### 3.7. Long Non-Coding RNAs (lncRNAs)

Differences in the expression of lncRNAs in osteosarcoma tissues compared to nearby non-cancerous tissues have been observed in microarray analyses (Figure 1). Some lncRNAs, including 91 H, BCAR4 and MALAT-1, are overexpressed, exhibiting oncogenic features, whereas others like Loc285194 and MEG3 are downregulated and serve tumor suppressive roles (Table 2). These lncRNAs exert an impact on biological processes and operate through various mechanisms, such as competing endogenous RNA (ceRNA) and modulation of the Wnt/β-catenin pathway [90].

HOTAIR and HOXD-AS1 are lncRNAs that promote tumorigenesis [91,92]. By interacting with the histone methyltransferase EZH2 (Enhancer of zeste homolog 2) at the *p53* gene promoter, EZH2 mediates the addition of methyl groups to histone H3 at lysine 27 and suppresses its transcriptional activity, thus affecting the tumor-suppressive role of p53 [92]. In addition, overexpression of the *TUG1* gene causes deregulation of miR-212-3p, causing lymph node metastasis, a larger tumor size and a decreased life expectancy for patients [93].

Some lncRNAs, such as SNHG1 and BCAR4, play significant roles in tumor progression through their interactions with specific microRNAs. SNHG1 is overexpressed in OS and targets miRNA–101-3p, suppressing its anti-oncogenic function. This downregulation of miRNA-101-3p leads to increased expression and activation of ROCK1 (Rho-associated protein kinase 1), a kinase that regulates cytoskeletal dynamics, cell adhesion and motility by phosphorylating several substrates such as GFAP, DAPK3, MYL9/MLC2, LIMK1, LIMK2, PFN1 TPPP and PPP1R12A. Activation of ROCK1 promotes cell migration, invasion and epithelial–mesenchymal transition (EMT), while SNHG1 also promotes tumorigenesis by inactivating the PI3K/AKT pathway lncRNA SNHG1 [94].

Another lncRNA, BCAR4, promotes tumorigenesis in OS by interacting with miR-1260a. miR-1260a levels are elevated in osteosarcoma tissues, but its expression is in opposition to BCAR4, depicting the direct interplay between BCAR4 and miR-1260a. miR-1260a mediates OS proliferation and migration after further knockdown of BCAR4 [95]. Thus, it is evident that SNHG1 and BCAR4 can have a therapeutic potential through distinct miRNA-dependent pathways.

### 3.8. Circular RNAs (circRNAs)

circRNAs are additional regulators of gene transcription and translation, acting as sponges for microRNAs (miRNAs), promoting tumorigenesis by functioning as competitive endogenous RNAs (ceRNAs) or binding and inhibiting miRNAs by blocking the miRNAs’ ability to carry out their tumor-suppressing roles. For example, circ_001422 was significantly upregulated in 40 out of 55 OS tissue samples compared to non-cancerous osteoblasts, with higher expression related to tumor size, advanced stage and metastasis (Table 2) [96]. Similarly, the circRNA cir-ITCHI was highly expressed in OS cell lines. Its overexpression enhanced OS cell growth, migration and invasion, while suppression increased miR-7 levels, restoring normal cell function [97]. circPVT1 is also increased in OS and promotes tumor progression. It functions by acting as a sponge for miR-423-5p, miR-205-5p and miR-26b-5p, affecting downstream signaling pathways and contributing to metastasis and chemoresistance [98]. Additionally, overexpression of circ_001569 has been associated with distant metastasis and advanced tumor stages in OS patients. In vitro experiments have demonstrated that increased circ_001569 induces resistance to cisplatin of OS cells by activating the Wnt/β-catenin signaling pathway [99].

Some circRNAs act as tumor suppressors by absorbing oncogenic miRNAs. CircTADA2A, a non-coding RNA expressed at high levels in both OS cells and tissue, acts as a sponge for miR-203a-3p regulating CREB3 activation, an identified OS-conducting gene. Inhibition of miR-203a-3p or CREB3 suppresses the functionality of CircTADA2A and thus the level of tumor malignancy [100]. circLARP4 is often found at lower levels in OS tissues compared to normal tissues, while high circLARP4 expression has been associated with increased tumor cell necrosis after resection and chemotherapy. It also acts as a sponge for microRNA-424 (miR-424) and enhances the chemosensitivity of OS cells to adriamycin and cisplatin [101].

Moreover, circRNAs, by interacting with miRNAs, control gene regulation in OS. A microarray-based circRNA study compared OS cells and normal cell lines and detected 12 expressed circRNAs with potential roles in cancer signaling pathways. Among these, hsa_circRNA_103801 was upregulated and targeted hsa-miR-370-3p, hsa-miR-338-3p and hsa-miR-877-3p, modifying pathways such as HIF-1, VEGF and angiogenesis. Contrastingly, hsa_circRNA_104980 targeted hsa-miR-660-3p and hsa-miR-1298-3p and was correlated with the tight junction pathway [102].

**Table 2:** lncRNAs and circRNAs implicated in OS pathogenesis.

lncRNA/circRNA	Role	Mechanism/Targeted miRNA	OS Phenotype	Reference
HOXD-AS1	Oncogenic	EZH2 recruitment, p53 suppression	Promotes metastasis, induces drug resistance	[92,103]
HOTAIR	Oncogenic	EZH2 recruitment, miR-126 sponge	Promotes EMT, increases resistance	[91]
TUG1	Oncogenic	miR-144/miR-132 sponge	Promotes metastasis and drug resistance	[93]
MALAT1	Oncogenic	miR-129 sponge leads to increased RET	Promotes migration, invasion	[90,104]
BCAR4	Oncogenic	miR-1260a	Promotes migration, invasion	[95,105]
MEG3	Tumor suppressor	p53 activation	Reduces proliferation, increases drug sensitivity	[106]
Loc285194	Tumor suppressor	miR-211	Reduces proliferation	[90,107]
circTADA2A	Oncogenic	miR-203a-3p	Promotes EMT and invasion	[100]
circPVT1	Oncogenic	miR-152-3p, miR-205-5p	Promotes metastasis and drug resistance	[98]
circ_001569	Oncogenic	miR-145	Increases MMP-9, AKT and drug resistance	[99]
circLARP4	Tumor suppressor	miR-424	Increases apoptosis, increases cisplatin sensitivity	[101]
circMTO1	Tumor suppressor	miR-630	Inhibits proliferation, migration and metastasis	[108]

Another study identified 20 circRNAs downregulated in OS, where circMTO1 was most significantly decreased. OS cells’ migration and invasion capacity was reduced after the introduction of circMTO1 into OS cells. Additionally, miR-630 was overexpressed in OS, followed by a decrease in its downstream target, the tumor suppressor *KLF6*. The overexpression of circMTO1 levels increased KLF6 levels, implicating circMTO1 in miR-630 activity, which then inhibits KLF6, facilitating tumor progression [108].

## 4. Epigenetic Modifications and Immune Cells in Osteosarcoma

**Figure 3:**
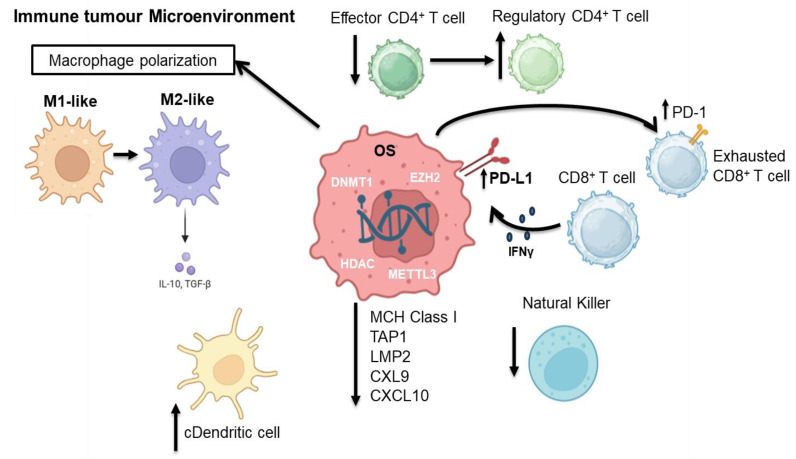
In the immune tumor microenvironment, OS cells exploit epigenetic modifiers—such as DNMT1, EZH2, HDACs and METTL3—to reprogram gene expression and facilitate immune evasion, in part through the upregulation of PD-L1. Macrophages shift from the M1 (anti-tumor and pro-inflammatory) to M2 (pro-tumor and immunosuppressive) phenotype, influencing inflammation or immune suppression by releasing IL-10 and TGF-β. Conventional dendritic cells (cDCs) present tumor antigens to CD4+ T cells, promoting their differentiation into either effector or regulatory subsets, and thereby modulating the immune response. Chronic antigen stimulation leads to functional exhaustion of CD8+ cytotoxic T cells, characterized by the expression of negative immune checkpoints such as PD-1. NK (natural killer) cells provide an additional tumor defense barrier. DNMT: DNA (Cytosine-5)-methyltransferase 1, EZH2: Enhancer of zeste homolog 2, HDAC: histone deacetylase, METLL3: methyltransferase-like 3, IFN-γ: Interferon Gamma, IL-10:Interleukin 10, TGF-β: Transforming Growth Factor Beta, NK Cell: natural killer cell, cDC: Conventional dendritic cell, CXCL9: C-X-C motif chemokine ligand 9, CXCL10: C-X-C motif chemokine ligand 10, LMP2: Low Molecular Mass Polypeptide 2, TAP1: Transporter Associated with Antigen Processing 1, MHC Class I: Major Histocompatibility Complex Class I, PD-1: Programmed Cell Death Protein 1, PD-L1: Programmed Death-Ligand 1. Created in https://BioRender.com. (accessed on 20 July 2025).

The tumor immune microenvironment (TΙME) of OS is composed mainly of T lymphocytes and macrophages, while there are also other subgroups such as B lymphocytes and mast cells. Low T cell infiltration is one of the immunosuppressive TIME characteristics of OS (Figure 3) [109]. Immune activity within osteosarcoma’s tumor microenvironment (TME) is shaped by epigenetic regulation and can affect tumor development and therapeutic resistance by modulating the cancer–immunity cycle, chemokine expression, antigen presentation and negative immune checkpoint expression [110]. Recent advances in AI have enabled Yu and colleagues to identify two distinct OS subtypes based on complex epigenetic signatures (*SFMBT2*, *SP140*, *CBX5*, *HMGN2*, *SMARCA4*, *PSIP1* and *CHD2*) exhibiting different immune TME profiles in respect to immune responses and sensitivity to immunotherapy [111]. Recent studies have shown that DNMT1 overexpression in OS cells downregulates MHC class I expression and antigen-processing genes such as *TAP1* and *LMP2* through promoter hypermethylation, impairing cytotoxic CD8+ T cell activation [112,113]. Li et al. have shown that DNMT1-mediated methylation of the C-X-C motif chemokine ligand 12 (*CXCL12*) promoter suppressed its expression by impairing T cell homing and increasing metastasis [114]. Yu et al. showed DNMT1-hypermethylation of pro-apoptotic genes, such as *TSSC3*, which contributes to immune escape [111]. Demethylating agents such as 5-aza-2’-deoxycytidine were reported to reverse these methylation effects and restore immune surveillance by enhancing CD8+ T cell responses [114].

Histone modifications, including deacetylation via the HDAC6/STAT3 pathway, have been shown to alter PD-L1 expression [115] and correlate with increased levels of CD8+ T cells and regulatory T cells (Tregs) [116]. Chemokine production, such as CXCL9 and CXCL10, required for effector T cell infiltration into tumors, is repressed due to histone modifications such as H3K27 trimethylation [117]. Moreover, the immunosuppressive TME is supported by Tregs and tumor-associated macrophages (TAMs), whose differentiation is also promoted by epigenetic enzymes such as EZH2 and DNMTs [118]. In addition, RNA modifications such as m1A and m6A methylation, along with regulation by non-coding RNAs (microRNAs, long non-coding RNAs and circular RNAs), affect macrophage polarization by promoting the M2-like phenotype [119], dendritic cell function, Tregs and myeloid-derived suppressor cells (MDSCs), and promoting an overall immunosuppressive TME composition [120]. Therapeutic targeting of epigenetic regulators can alter immune cell functions and overcome TME-mediated immunosuppression, offering new approaches to treatment effectiveness.

## 5. Epigenetic Therapeutic Targets in OS

**Figure 4:**
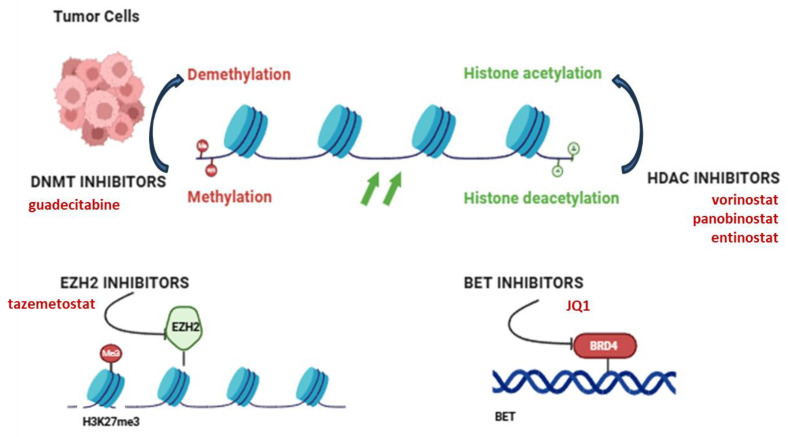
Overview of current epigenetic drug targets and their mechanisms of action. Four main classes of epigenetic inhibitors, DNMT, HDAC, EZH2 and BET, have been shown to act on chromatin remodeling and gene expression in OS cells. These therapies target DNA methylation, histone acetylation/deacetylation, histone methylation and bromodomain transcription, respectively, to reverse cancer-associated epigenetic changes. DNMT: DNA methyltransferase, HDAC: histone deacetylase, EZH2: Enhancer of zeste homolog 2, BET: Bromodomain and extra-terminal domain. Created in https://BioRender.com (accessed on 19 July 2025).

In OS with high metastatic potential, current targeted treatments are not particularly efficient in eradicating the primary tumor and have failed to bring significant results in Phase 2 clinical trials. Therefore, there is a high demand for robust preclinical data and identification of novel molecular targets to be included in the standard methods of treatment to avoid metastatic regression [121]. Current research progress on epigenetic mechanisms and related treatment modalities have been explored in preclinical and clinical settings with promising outcomes. DNMT inhibitors, along with HDAC and EZH2 inhibitors and BRD4 blockers, have been investigated in OS in vitro and in vivo models with significant results (Figure 4).

The histone methyltransferase NSD2 (nuclear receptor binding SET domain protein 2) was detected to be upregulated in OS tissues. *NSD2* gene knockdown increased apoptosis of OS cells and reduced H3K36me2 levels at *SOX2* and *BCL2* gene loci, rendering OS cells more sensitive to cisplatin. Extracellular signal-regulated kinase (ERK) and AKT signaling pathways were reversed upon *NSD2* knockdown, indicating NSD2 as a good research target to overcome chemoresistance in OS. However, many core subunits and co-factors that are involved in the underlying mechanism of NSD2 activity are still unknown. Nevertheless, NSD2 may present a potential therapeutic target in combined chemotherapy and a good prognostic biomarker in OS [122]. HDAC inhibitors such as vorinostat and panobinostat have been shown to increase apoptotic and immune gene expression, inhibiting proliferation and rendering OS cells more susceptible to immunotherapy and chemotherapy (Table 3). However, these inhibitors have a wide range of actions, resulting in mild in vivo efficacy in bone tumor models and systemic side effects [123]. Their limited use as monotherapy indicates the necessity of treatment combinations. EZH2 inhibitors have shown that reverse gene silencing suppresses tumor growth and potentially reprograms the immune TME in an OS animal model and cell lines, by reducing the number of immunosuppressive TAMs and Tregs and restoring CXCL9 and CXCL10 expression. However, as EZH2 also controls osteoblast differentiation, its inhibition may affect normal bone remodeling, which is rather critical in pediatric patients [117,124].

Another study has investigated the therapeutic application of DNA demethylation in OS. Gene ontology analysis revealed several enriched genes that are involved in hormone metabolism (*STC2*, *HSD17B8* and *CYP1B1*), neurological function (*ADRA1A*, *TFAP2A*, *CYP1B1* and *MBP*) and skeletal morphogenesis (*HOXA6*, *TFAP2A* and *HOXC8*) to be silenced by methylation in OS. Six of these genes were osteogenesis-related and five of them tumor suppressors. Upon treatment of three OS cell lines (HOS, MG63 and 143B) with 5-aza-deoxycytidine (a nucleic acid synthesis inhibitor that is used as medication for myelodysplastic syndromes) in a dose-dependent way, a reduction was observed in cell viability. In vivo studies using 143B and HOS tumors showed reduced tumor volumes in HOS cells by 65.0% and 29.3% upon administration of 1 and 2 mg/kg 5-aza-dC, respectively, for three weeks. In 143B cells, tumor volumes were reduced by 94.0% and 43.1% upon administration of 1 and 2 mg/kg 5-aza-dC for 4 weeks, respectively. Necrosis was increased by the treatment and lung metastases decreased, but without reaching statistical significance. Overall, these results point towards the efficacy of 5-aza-dC treatment in OS and the need for further investigation.

Furthermore, the effects of decitabine (5-aza-dC) and VPA (valproic acid) were investigated in OS, Saos2 and MG63 cell lines in an effort to elucidate the molecular mechanism that underlies stemness maintenance. Key findings of this preliminary study include the significant effects of VPA and DAC inducing CSCs’ expansion, as well as in characterizing the epigenetic profile of CSCs which involves DNA hypomethylation, H3K3me2 and H3K4me3, increased H3 histone acetylation and decreased H3K9me3 and H3K27me3 levels. Moreover, DNMT3a and HDAC2 enzymes were shown to be involved in the cancer stem phenotype in OS, and HDAC2 expression was suggested as a therapeutic target. Elucidation of specific epigenetic events that maintain the undifferentiated state of cells is of primary importance and enables a better design of therapeutic strategies and epigenetic drugs in combination with conventional chemotherapy [77,125,126].

Notably, demethylating agents like 5-aza-dC can restore GADD45 as well as CREG1, highlighting their therapeutic potential. Decitabine functions as a hypomethylating agent by forming irreversible covalent adducts with DNA methyltransferases, leading to their degradation. At high concentrations, decitabine induces cell apoptosis in cancer cells, while at lower concentrations, its anticancer effects are primarily driven by DNA methylation [127]. However, their use remains limited due to low specificity, systemic toxicity and low bone penetration. Additionally, as observed in other cancer types, hypomethylation may trigger immune responses or activate oncogenes [127,128]. The best dosage and delivery methods are under investigation, and their effectiveness in OS is still preclinical.

Currently, RNA methylation inhibitors are evaluated as therapeutic targets (Table 3). Experimental studies have shown that METTL3 can promote OS progression through DRG1 regulation and NF-κB/STAT3 signaling through m6A. In vitro studies showed that METTL3 inhibition impaired OS cell viability [129]. However, METTL inhibitors have not yet been clinically tested.

Given that epigenetic agents as monotherapies have not given the expected results, their combination with conventional treatments has emerged as a promising therapeutic strategy in OS. HDAC and DNMT inhibitors might sensitize resistant OS cells to doxorubicin or cisplatin by reactivating apoptotic pathways (Table 3). Preclinical studies demonstrated that the combinations of 5-aza-dC with cisplatin or VPA have synergistic anti-tumor effects, including reduced tumor burden and decreased lung metastases [128,130].

Moreover, emerging evidence suggests that immunotherapies, such as anti-PD-1 and anti-CD47, combined with epigenetic inhibitors could have a synergistic effect against OS. Studies have shown that decitabine-induced upregulation of antigen presentation genes increased the efficacy of the anti-PD-1 treatment when combined in other solid tumors, suggesting a similar effect in OS [118,128]. DNMT and HDAC inhibitors can increase tumor immunogenicity by enhancing antigen presentation and the expression of “eat me” signals, thereby enhancing the immune surveillance of the tumor cells. Blocking the “don’t eat me” signal with the CD47 antibody facilitates the phagocytosis of cancer cells by the macrophage, eliminating and in turn, preventing metastasis in preclinical OS models. Recent studies by Roudi et al. demonstrate that CD47 blockade not only suppresses tumor progression but its efficacy is enhanced when combined with agents capable of modifying the tumor epigenome, as these drugs can convert immunologically “cold” tumors to “hot,” promoting increased immune cell infiltration and responsiveness [131]. Ahvati et al. stated that targeting epigenetic modifications reprograms both the tumor cells and their TME, facilitating the effectiveness of immunotherapies like anti-CD47 in OS [132].

**Table 3:** Therapeutic targets/trials on epigenetic modifications in osteosarcoma.

Epigenetic Modification	Target	Role in Therapy	Emerging Therapies/Clinical Trials	References
DNA Methylation	DNMTs (DNMT1/3A/3B)	DNMT inhibitors restore silenced tumor suppressors	Guadecitabine in preclinical OS models; synergistic effects with chemotherapy	[127]
Histone Modifications	HDACs	Reactivate silenced genes; reduce metastasis and resistance	Vorinostat, panobinostat and entinostat in phase I/II trials (NCT04308330)	[123]
	EZH2 (HMT)	Mediates H3K27me3 silencing; promotes progression	Tazemetostat tested in pediatric sarcomas including OS (NCT02601950)	[133,134]
	KDM6B (JMJD3)	Demethylates H3K27me3; upregulation linked to metastasis	Experimental inhibitors under development	[135,136]
	LSD1/KDM1A	Demethylates H3K4me1/2; promotes proliferation	ORY-1001 (iadademstat) in hematologic/solid tumors (exploratory for sarcoma)	[137]
Non-coding RNAs	Oncogenic lncRNAs/miRNAs (e.g., THAP9-AS1)	Modulate epigenetic silencing via DNMT recruitment or miRNA targeting	Development of miRNA mimics (e.g., miR-34a) and anti-lncRNA ASOs	[138]
RNA Modifications	METTL3, FTO, ALKBH5	Alter mRNA stability/translation; affect immune regulation	Targeted inhibitors in discovery (e.g., STM2457 for METTL3)	[129]
Epigenetic Modifier Genes	DDX24, HDAC4, SP140, UHRF2, etc.	Involved in gene regulation and prognosis; predict therapy response	Incorporated into risk scoring systems for immunotherapy stratification	[139]
Enhancer Elements	Metastatic enhancer regions	Control metastasis-related gene expression	BET inhibitors (e.g., JQ1) show promise in blocking enhancer activity	[140]

## 6. Conclusions and Future Perspectives

Altogether, OS is considered a cancer of multifactorial origin, implicating a wide range of interactions between genetic and epigenetic factors that can cause the deregulation of signaling pathways and destructive bone tissue physiology. It has a poor prognosis, and the conventional treatment is limited to surgical resection of the tumor combined with chemotherapy or radiotherapy. However, its recurrence and chemotherapy resistance have such high frequency that they often lead to decreased survival time. Nowadays, with the knowledge of the importance of epigenetic modulation in cancer progression, immune evasion and diagnosis, the introduction of new therapeutic agents that target epigenetic alterations—particularly through DNMT, HDAC and EZH2 inhibitors—might present a new strategy to tackle the chemotherapy resistance in OS and even reduce the recurrence rate. Every year, more studies become available, providing new evidence on epigenetic modifications (DNA methylation, histone modifications and the non-coding RNAs), suggesting that these mechanisms can act separately or in concert to alter the transcriptional level of oncogenes, and even deregulate the signaling pathways leading to oncogenesis (Figure 2).

Significant advances over the last years, enriched by new multi-omics technologies and the development of new experimental models of tumors with OS behavior, have attracted immense scientific interest in finding ways to interfere with these epigenetic mechanisms and detect novel approaches for treatment, better diagnosis and even prognosis when there is gene predisposition. The integration of artificial intelligence (AI) and machine learning in OS research enabled the identification of complex epigenetic signatures and the discovery of predictive epigenetic biomarkers, facilitating patient stratification and personalized treatments. Notably, recent studies using AI identified OS predictive biomarkers by detecting two key molecules, SERPINE2 and CPT1B, that were positively linked to memory B cells and CD8+ T cells, respectively [141]. Li et al. have utilized AI to construct epigenetic modification-related gene prognostic signatures (EMRPSs), consisting of *DDX24*, *DNAJC1*, *HDAC4*, *SIRT7*, *SP140*, and *UHRF2*, to predict response to chemotherapy and immunotherapy [139,142], underscoring the potential of precision medicine in OS management. Moreover, Yin et al. used AI-built risk models on epigenetic profiles in order to stratify patients for optimal therapy selection [143]. Other studies underline the collective role of the TME along with other therapies [144,145]. In the near future, it is expected that more advances will be achieved and combined therapeutic schemes will be employed. Despite these advances, the mechanisms by which specific epigenetic modifications orchestrate immune evasion and resistance to therapy in OS require further elucidation. While AI-based biomarker discovery holds great promise, prospective clinical validation and standardization are necessary.

A deeper understanding of OS epigenetic modifications is therefore demanded, coupled with innovative therapeutic strategies and AI-driven biomarker discovery, to overcome current challenges and improve outcomes for patients with OS.
